# A Mobile-Based Tailored Recommendation System for Parents of Children with Overweight or Obesity: A New Tool for Health Care Centers

**DOI:** 10.3390/ejihpe10030057

**Published:** 2020-08-07

**Authors:** Lisa Afonso, Rui Rodrigues, Joana Castro, Nuno Parente, Carina Teixeira, Ana Fraga, Sandra Torres

**Affiliations:** 1 Faculty of Psychology and Educational Sciences and Center for Psychology, University of Porto, Rua Alfredo Allen, 4200-135 Porto, Portugal; storres@fpce.up.pt; 2 Faculty of Engineering, University of Porto, and INESC TEC, Rua Doutor Roberto Frias, 4200-465 Porto, Portugal; rui.rodrigues@fe.up.pt; 3 Maia-Valongo Health Centre Group, Avenida Luís de Camões, n.º 290, 3.º Andar, 4474-004 Maia, Portugal; joanacastro@hotmail.it (J.C.); nunomiguelpm@gmail.com (N.P.); carina.sbt@gmail.com (C.T.); anafraga.med@gmail.com (A.F.)

**Keywords:** e-health, guideline adherence, healthy lifestyle, children, obesity

## Abstract

Childhood obesity is associated with unbalanced lifestyle patterns, and new strategies are needed to support parents in the compliance with the guidelines for children’s age. Tailored automatic recommendations mimic interpersonal counseling and are promising strategies to be considered for health promotion programs. This study aimed to develop and test a mobile recommendation system for parents of preschool children identified with overweight/obesity at health care centers. Evidence-based recommendations related to children’s eating, drinking, moving, and sleeping habits were developed and tested using a questionnaire. A pilot study was conducted in a health care center to test how using an app with those tailored recommendations, in video format, influenced parents’ perceptions of the child’s weight status and their knowledge about the guidelines, compared to a control group. The chi-squared test was used for categorical variables and the Mann–Whitney U test for continuous variables (*p* < 0.05). A high proportion of parents were already informed about the guidelines, but their children were not meeting them. After watching the tailored recommendations, there was an increased knowledge of the guideline on water intake, but there was no improvement in the perception of the child’s excessive weight. Parents may benefit from a mobile-based tailored recommendation system to improve their knowledge about the guidelines. However, there is a need to work with parents on motivation to manage the child’s weight with additional strategies.

## 1. Introduction

Obesity remains one of the most common and complex health conditions worldwide. In 2017, over 38 million children under the age of five lived with overweight or obesity [1]. Childhood obesity is frequently preceded by unbalanced lifestyle patterns, such as an unhealthy diet and insufficient physical activity (PA). The earlier these behaviors can be altered, the better the chance of healthy physical development and its maintenance until adulthood. In fact, the preschool age is especially important to guarantee healthy growth because better energy balance at this age may contribute to a delayed adiposity rebound, which is associated with a lower risk of future excessive weight [2]. To prevent and treat childhood obesity, family-based lifestyle interventions are recommended [3,4]. It is also recommended to design programs that incorporate a combination of key lifestyle factors related to excessive weight, namely, by promoting healthy eating and drinking patterns; encouraging the consumption of fruit, vegetables, and water; avoiding high-fat/high-sugar foods and sugar-sweetened beverages [3,4,5,6]; as well as promoting healthy PA [7] and sleeping habits [8,9].

Well-child visits at health care centers are ideal opportunities to detect deviations in weight trajectories and improve family habits. However, there are restrictions on time, resources, and knowledge that limit the ability to comprehensively counsel parents and properly enable and motivate them to adhere to healthy lifestyle guidelines [10,11,12]. Therefore, parents are usually counseled on a few lifestyle topics based on the dissemination of information regarding the recommendations for the child [13,14]. It would be crucial to also include well-studied behavior change techniques when counseling parents, such as promoting environmental restructuring [15]. Interventions to promote healthy behavior based on behavior change theories, particularly those grounded on the theory of planned behavior (TPB), have been associated with better outcomes compared with non-theory-based interventions [16]. According to the TPB, people may fail to accomplish a behavior because they lack resources, opportunities, or skills, and, therefore, they do not have control over it [17]. The TPB suggests that the intention to perform a behavior is predicted by attitudes, subjective norms, and perceived behavioral control [17]. In addition to providing information to parents about the guidelines, it would be important to work with them on practical skills to perform the behavioral change [17,18]. In relation to the management of children’s food intake, those strategies are called “food parenting practices”. Previous studies have indicated that when parents decide to control their child’s weight, they often use coercive controlling practices (such as pressure to eat vegetables and overt restriction of energy-dense foods), which have been shown to be counterproductive [19,20]. A recent compilation on food parenting practices by Vaughn et al. proposed alternatives to controlling practices, namely, practices of structure (e.g., changing the availability of foods at home) and autonomy support (e.g., nutrition education) [21]. These constructs are aligned with self-determination theory (SDT) [21,22], as they aim to promote decision-making skills in children, developing in them a sense of ownership and endorsement of their behaviors and, ultimately, self-regulation in eating.

Technology-based solutions, such as computer and mobile applications, are promising for health care promotion, as they can include interactive and personalized components to engage users [3]. Tailored recommendations conveyed through those solutions have been shown to be effective in health education programs covering a wide range of behaviors [23]. These recommendations mimic interpersonal counseling, where they return only the necessary information to the user profile in an automated way [23]. Knowledge-based filtering may be an appropriate technique for tailored digital health programs [24]. It works by predicting items based on explicit knowledge about users (e.g., via questionnaires) and is able to predict items that are relevant and tailored to the user’s interest. Therefore, this filtering would possibly enhance the efficiency and effectiveness of the health care recommendations [24].

Based on this, an evidence-based tailored recommendation system was developed as a mobile application (app) in order to target parents of children aged 3–6 years who were identified in health care centers as having overweight/obesity. It was intended to be a TPB evidence-based system, in which the recommendations to parents were structured according to different predictors of behavior, i.e., beliefs, attitudes, and perceived behavioral control. The recommendations promoted positive food parenting practices in line with SDT and in expectation of an improvement in parents’ perceived behavioral control over their child’s eating behaviors. A pilot test in a health care center was designed to test the procedures of a randomized controlled trial (RCT). This study aimed to describe the effect of its use on changing parents’ knowledge about the guidelines related to children’s lifestyle, as this can be seen as a short-term outcome that represents a potential mechanism of efficacy of the designed intervention [17,25]. This study aimed to indicate barriers to acceptance and adherence to this e-health intervention in order to perform potential adjustments before running the RCT in health care centers for the prevention and treatment of overweight/obesity.

## 2. Materials and Methods

### 2.1. Study Design

In order to tailor recommendations, an automated filtering system was designed based on parents’ answers to a questionnaire that evaluated (a) their knowledge of the guidelines for the child’s age (about eating, drinking, moving, and sleeping habits), (b) their perception of the child’s weight, and (c) the children’s behaviors (eating, drinking, moving, and sleeping habits). This questionnaire was included in an Android app as well as recommendations for each topic through videos that were recommended to parents according to the needs identified in the questionnaire. This app, called Fammeal, was part of a system developed by the research team.

The recommendation system was tested by (a) the application of the questionnaire that tailored recommendations to analyze the percentage of parents who would be recommended to watch videos on each topic and (b) describing how using the app with the recommendation system affected parents’ beliefs about the guidelines for children with overweight/obesity compared to a control group.

### 2.2. Recommendation System Development

#### 2.2.1. Defining Recommendations for Parents

The decision on recommendations to parents was supported by the need to raise awareness of the child’s excessive weight, assuming that this factor is an important determinant of the parent’s motivation to improve the family environment [26]. Thus, this issue was the first addressed. The recommendations that were given to parents focused on the promotion of key lifestyle factors identified in the literature as relevant to be included in programs to prevent and treat childhood obesity [3,4], namely, healthy eating, drinking patterns [3,4,5,6], PA [7], and sleeping habits [8,9].

For each topic, parents received recommendations related to some dimensions of the TPB [17,18]. The TPB suggests the intention to perform a behavior (i.e., the individual’s conscious plan or decision to exert effort in order to engage in a particular behavior) is predicted by [17,18]:

- Attitudes, i.e., one’s evaluation of performing an action, highly determined by beliefs related to the behavior;
- Subjective norms, i.e., one’s belief about social expectations; and
- Perceived behavioral control, i.e., one’s perception of the degree of ease and difficulty of the behavior.

The recommendations were based on some dimensions of this model, namely:

1. Parents’ perceptions about the children’s weight status and knowledge about the guidelines for their age as determinants of their behavioral beliefs and, consequently, of their attitudes toward the behavior;
2. The importance attributed to their children’s weight status and the guidelines for their age, reflecting their attitudes; and
3. Practical strategies to improve those outcomes and to improve perceived behavioral control.

The practical strategies to improve food intake were food parenting practices recommended in the map by Vaughn et al. [21]. The aims of each recommendation for each lifestyle component are represented in Table 1.

#### 2.2.2. Defining the Tailored Recommendation System

To tailor recommendations, a questionnaire was developed (Table 2) to survey parents’ knowledge about their children’s weight status (question 1a) and their concern about it (question 1b); the lifestyle guidelines regarding their child’s dietary intake (Question 2a), PA, and sleeping habits (question 3a); and their children’s behaviors related to those lifestyles (Questions 2b and 3b). The cutoffs for the guidelines (see Table 2) were used as the condition for receiving a recommendation. Parents’ knowledge being discordant with the guidelines defined the attribution of recommendations related to beliefs, while parents’ unconcern about their child’s weight or behaviors being discordant with the guidelines led to recommendations related to attitudes and perceived behavioral control.

#### 2.2.3. Development of the App

A system that consisted of an Android app, called Fammeal, for parents of children with overweight/obesity attending health care centers was developed. To protect the participants’ personal data, a personal ID was generated to be introduced by parents into the app during the registration process. That ID matched the one used to identify the participant in the study database, where personal data were stored, complying with the data protection guidelines. Additionally, no personal information was saved or removed from the device.

**Figure 1:**
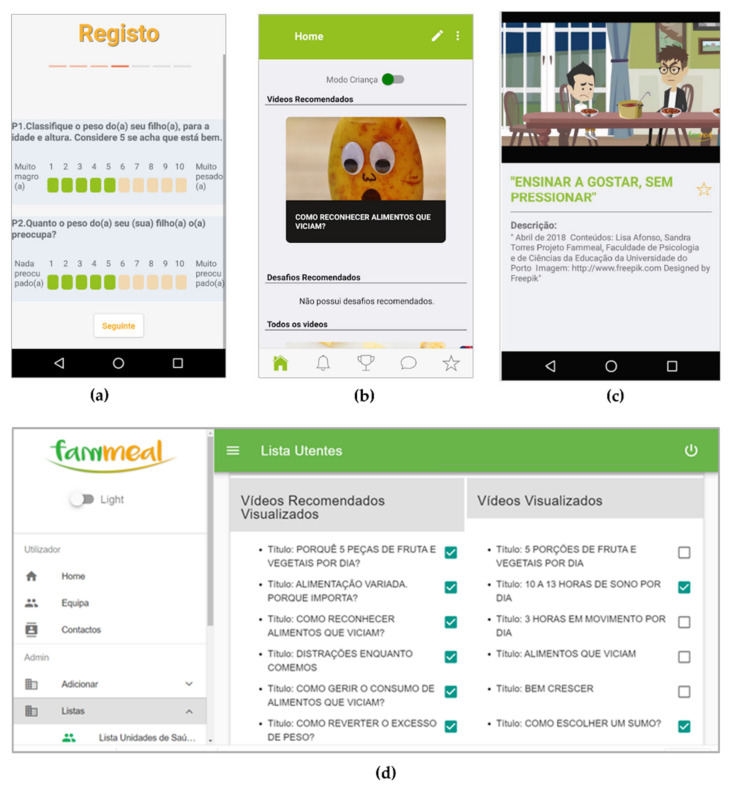
Fammeal app and monitoring website screenshots: (**a**) app—Registration questionnaire; (**b**) app—Home page; (**c**) app—Video; (**d**) monitoring website—Checklist with “recommended” videos on the left and all the “other” videos on the right (with a check mark whenever watched).

The app included the recommendation system, i.e., the questionnaire to be filled out during the registration process (that took no more than 15 min to complete), the cutoffs to tailor recommendations, and educational videos that appeared automatically as “recommended” or as “other videos” (users could access all the videos) based on parents’ answers to the questionnaire. A website to store the videos was created, and, as the user accessed the video in the app, it connected with the website via the Internet, and the video could be watched in the app (Figure 1) [27].

This website included private access for the administrator with monitoring tools that displayed parents’ usage reports, namely, their answers to the registration questionnaire and a checklist with the videos that were recommended and those watched by parents (“recommended” and “other” videos).

Scripts for 15 videos were developed (Table 3).

Each video targeted one dimension of the TPB previously described. The exceptions were the videos related to PA and sleep, which promoted more than one dimension of the TPB. Because the tailoring system was based on self-reporting, which is subject to socially desirable responding [28], three videos (Table 3: Videos 3, 5, and 9) were defined as default on the list of recommended videos, regardless of parents’ answers to questionnaires.

Parents were recommended to watch 8–10 videos (three of which were recommended by default). If parents had more than 10 videos recommended by the system, then some were randomly excluded, with the exception of the five videos related to beliefs, which were defined as priorities (one for each lifestyle topic) because this is the first step to promoting change according to the TPB. If less than eight videos were recommended to parents, then the remaining videos were randomly selected to fill the eight videos required.

The app also included gamification strategies in order to motivate parents’ adherence, which are described in another scientific manuscript.

### 2.3. Testing of the Recommendation System

To test the recommendation system, two studies in a health care center in Porto, Portugal, were developed over three months. Parents of preschool children were invited by medical doctors to participate during the annual well-child care visit. These studies were addressed using different samples.

In Study 1, medical doctors invited parents to answer the questionnaire developed by the team to tailor recommendations, independently of the weight status of their child. This study intended to describe the adequacy of the recommendation system to parents of children of this age range.

In Study 2, medical doctors invited parents of children with overweight/obesity to participate in the pilot study. This pilot study was registered prior to the enrollment of participants [29]. Due to the pilot nature of this study, no sample size calculations are provided. However, a minimum of 12 participants has been reported as enough to estimate average values and variability in pilot studies with continuous variables [25]. Due to the small sample size, data analyses were mainly descriptive and exploratory. This study intended to describe parents’ perceptions regarding their child’s weight status and their knowledge about the guidelines in order to monitor the recommended videos that they watched in the app and the changes in their perceptions and knowledge afterwards and to compare these to the control group.

#### 2.3.1. Participants

Participants were parents/caregivers of children between 3 and 6 years of age. To participate, they should be involved in the child’s feeding management (≥5 for involvement on a 0–10 rating scale, from “not at all” to “extremely involved”, regarding food acquisition and meal planning and preparation). In Study 1, participants were selected regardless their child’s weight status. In Study 2, participants were invited to participate if they simultaneously:

1. Were parents of children with overweight/obesity for their age (according to the World Health Organization criteria [30]);
2. Had access to an Android device with an Internet connection;
3. Were interested in participating in two interviews in the health care center; and
4. Were willing to install the app and use it for four weeks.

For both studies, parents of children with any medical conditions that affected growth, intake, or PA or with any professional dietary advice in the previous six months were excluded.

Informed consent was obtained from all participants. This study complied with the ethical principles of the Declaration of Helsinki [31] and was approved by the Ethics Committee of the Faculty of Psychology and Educational Sciences of the University of Porto (reference number: 2017/10-4). This study also complied with data protection principles, and the Portuguese Data Protection Authority approved the study (reference number: 14441/ 2017).

#### 2.3.2. Study 1—Test of the Adequacy of the Recommendation System

In Study 1, 35 parents were invited to answer the questionnaire after the well-child care visit with the help of the project technician. All parents invited agreed to participate. The questionnaire was developed by the team to tailor recommendations (Table 2). The exception was food intake, which was evaluated using a more accurate method, namely, a qualitative food frequency questionnaire, administered by a trained researcher [32]. Parents reported the usual frequency of intake of fruits and vegetables, sugar-sweetened beverages, and energy-dense foods, from “never” to “more than four times a day”, for a list of foods. The food frequency resulted from the sum of frequencies of foods that composed each group.

#### 2.3.3. Study 2—Pilot Study

**Figure 2:**
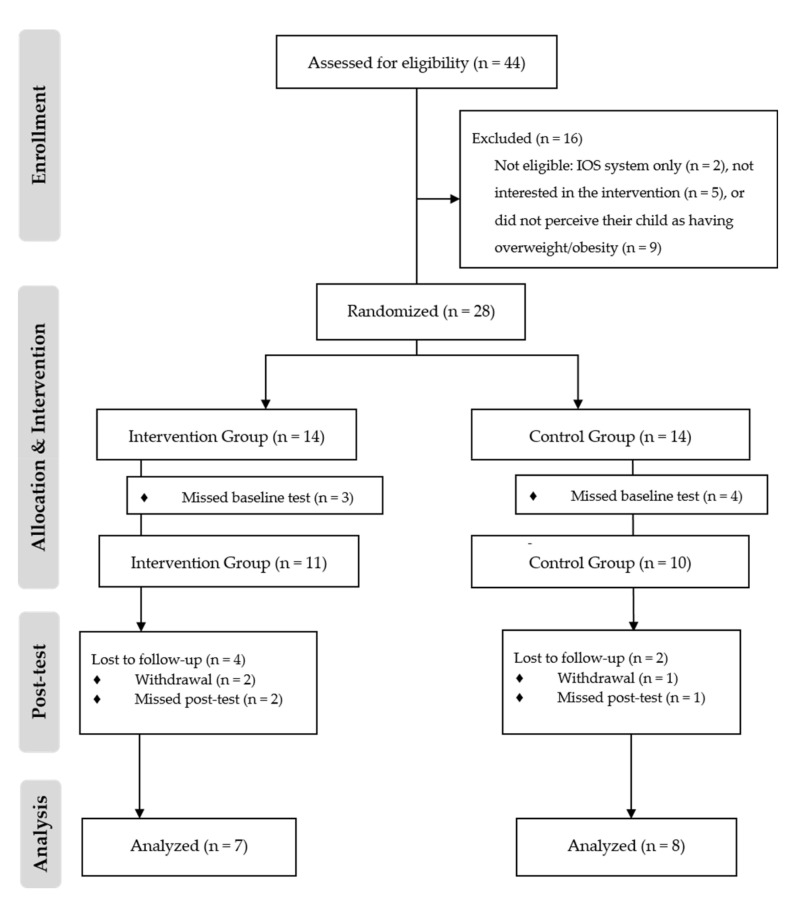
Flowchart of the pilot study.

This was a pilot randomized controlled trial with a parallel assignment: a 1:1 ratio of the intervention and control group. Medical doctors invited 44 parents to participate. To encourage parents to participate, medical doctors explained why the child was selected and what the consequences of having too much weight at this age range are. They also raised awareness about the need to improve family lifestyle habits in order to reduce their child’s excessive weight. Additionally, medical doctors explained to parents that the intervention allowed for flexibility, as they could use the app in accordance with their schedules. Of those invited, 28 agreed to participate (63.6%). The main barrier to adherence was the parents’ denial of their child’s excessive weight: nine of the invited parents (n = 9; 20.5%) refused to participate because they reported that they still did not perceive their child as having overweight/obesity, even after an explanation from the medical doctor. Five parents (11.4%) reported that they were not interested in the intervention (without specifying the reasons), and two (4.5%) did not have an Android device to use in the intervention (iOS system only). Parents that agreed to participate were randomized by the block randomization method into either the control or the intervention group using the random number generator in Excel. Of those, 21 attended to the baseline assessment, where they completed the questionnaire (see Table 2). Parents in the intervention group (n = 11) were provided with the app and invited to use it for a four-week period. Parents in the control group (n = 10) were treated as usual, namely, by receiving recommendations to improve the family lifestyle in the well-child care visit given by the medical doctor. Fifteen parents attended the post-test assessment. The flowchart of the pilot study is depicted in Figure 2.

### 2.4. Statistical Analysis

In Study 1, to test the adequacy of the recommendation system, each variable was transformed into a binary classification according to the cutoff value determined to receive a recommendation. The proportion of parents that would receive recommendations on each topic was evaluated as well as the percentage of children that failed to meet the guidelines, regardless of parents’ knowledge about the guidelines.

In Study 2, due to the small sample size, we performed an analysis of change, comparing the change scores between groups before and after the intervention. The change scores were calculated by post-test scores minus baseline scores. Data normality was tested using the Kolmogorov–Smirnov test. The chi-squared test was used for categorical variables (comparison of group characteristics at the baseline). Regarding the analysis of change, due to a lack of normal distribution of the scores and the possibility of a strong ceiling effect, the Mann–Whitney U test was used (*p* < 0.05). All statistical analyses were performed with SPSS v24 [33]. The magnitude of the difference in the knowledge between the baseline and post-tests was described by the effect size, represented by the coefficient of determination (*r*^2^). The cutoffs considered for a small, medium, and large effect size were 0.20, 0.50, and 0.80, respectively [34].

## 3. Results

### 3.1. Participants

Participants’ characteristics are described in Table 4. Characteristics for Study 2 refer to the baseline (n = 21). Participants allocated to the intervention group did not differ significantly from those in the control group prior to the intervention (baseline) in sex, age, and education (*p* > 0.05).

### 3.2. Study 1—Testing the Adequacy of the Recommendation System

Table 5 presents the percentage of parents who were discordant with, not concerned about, or whose children were not meeting the guidelines and, thus, would receive recommendations according to the cutoffs considered in the recommendation system. The majority of parents knew the guidelines for energy-dense food intake (n = 34; 97.1%), sugar-sweetened beverage intake (n = 35; 100.0%), and PA time (n = 30; 85.7%).

Among parents aware of the child’s excessive weight or the guidelines (i.e., those not eligible to receive recommendations in 1a, 2a, and 3a, in Table 5), a relevant proportion was not concerned about it, or their child was not meeting the guidelines:

1. Regarding weight status, of the three parents who perceived their child’s excessive weight, one (33.3%) was not concerned about it.
2. Considering only parents who knew the guidelines, 55.8% of parents (n = 19) reported that their children ate more energy-dense foods, 25.7% (n = 9) that their children drank more sugar-sweetened beverages, and 13.3% (n = 4) that their children moved less than recommended.
3. From those parents that knew the guidelines for fruit and vegetable intake (n = 22), 63.6% (n = 14) reported that their children ate less than recommended.
4. Regarding water intake, considering only parents who knew the guidelines (n = 5), 60% of the children (n = 3) failed to meet them.

### 3.3. Study 2—Pilot Study

In the baseline test, all parents (n = 15) were aware of the guidelines for the intake of energy-dense foods and sugar-sweetened beverages and for sleep and PA time. Despite this, 73% of parents (n = 11) reported that their children ate more energy-dense foods, 33.3% (n = 5) that their children drank more sugar-sweetened beverages, 40.0% (n = 6) that their children moved less, and 6.7% (n = 1) that their children slept less than recommended.

Parents in the intervention group only received recommendations to watch videos related to beliefs concerning their perceptions about their child’s weight and related to the guidelines for fruit, vegetable, and water intake. Therefore, only the change in parents’ perceptions and knowledge about the guidelines for those topics was determined (Table 6). The monitoring reports allowed for confirming that parents in the intervention group watched all the recommended videos and that they watched a mean of three extra videos beyond those that were recommended.

A significantly greater increase in the knowledge scores for water intake (U = 0.0, *p* < 0.001; large effect size) was found for the intervention group than for the control group. No significant differences between the intervention and control group were found for perceptions about the child’s excessive weight and for knowledge about the guidelines for fruit and vegetable intake (*p* > 0.05).

## 4. Discussion

A mobile recommendation system for parents of preschool children with overweight/obesity was developed and tested. Of all the planned recommendations, the most needed recommendation was the one related to the guidelines for water intake. A high awareness of parents toward the guidelines for the children’s age was found, especially regarding energy-dense foods, sugar-sweetened beverages, and PA. In Study 2, that awareness was even more pronounced, with all parents knowing those guidelines. As Study 2 only included children with overweight or obesity, this may indicate that those parents have already been informed due to the weight condition of their children. However, a high percentage of the children of informed parents had failed to meet those guidelines, especially concerning energy-dense foods. These results were observed in other studies, which found satisfactory knowledge of parents related to the child’s excessive weight but low compliance with the guidelines [35]. This may be related to difficulties and barriers that parents may face, such as low management over children’s environments beyond the home or children’s high interest in energy-dense foods [36]. This reinforces the need to give recommendations that may contribute to improving parents’ perceived behavioral control on those topics, namely, by promoting positive food parenting practices [13,14]. The system developed included recommendations on those topics (not tested in this pilot study) to contribute to improving parents’ ability to change the child’s eating habits. Additionally, a high percentage of parents underestimated the child’s excessive weight, which is consistent with the previous literature [37].

A pilot study with the mobile recommendation system was carried out with parents of children with overweight/obesity. That study aimed to describe the change in parents’ knowledge about the guidelines for the child’s age and their perception of their child’s weight status after using the recommendation system in an app, compared to a control group. The main barrier to adherence was the parents’ denial of their child’s excessive weight: 20.5% of parents refused to participate because they reported that they still did not perceive their child as having overweight/obesity, even after an explanation from the medical doctor. Even after accepting to participate and after the medical doctor’s explanation, 40% of the parents continued to not recognize the child’s excessive weight in the baseline test. Additionally, the parents who were in the intervention group still did not recognize their child as having overweight/obesity in the post-test, even after watching the video about healthy growth, which explained what deviation from the normal percentile means as well as the short- and long-term consequences of their child’s weight status. This means that, in order to change parents’ beliefs about their children’s weight status, it is not enough to provide them with knowledge.

After watching all the recommended videos, there was an increased knowledge of the guidelines for water intake, the topic identified as the most in need of recommendation. This reinforces that the system can be useful to inform parents about lesser-known lifestyle guidelines. Strategies employed in this intervention, such as using an app with recommended videos, may have led to higher engagement of parents because all the parents in the intervention group watched their recommended contents. However, this may not be enough to make them aware that the children need to change their lifestyle because they first have to recognize the importance of changing the child’s weight status [26]. This can also explain the low compliance with the guidelines despite their knowledge about them. The fact that this intervention was targeted to children with overweight and not only to children with obesity may make it difficult for parents to recognize their excessive weight. Studies indicated that the parents only recognized a child’s excessive weight if there was a substantial deviation in the child’s body size from perceived normality, especially if they were between the ages of 2 and 6 years old [37,38]. This indicates that new methodologies to motivate parents to manage their child’s weight have to be considered. One hypothesis would be to facilitate at least one session of motivational interviewing because it showed positive effects in the improvement of behaviors in parent–child health interventions, including those related to excessive weight [39]. This may be included in an e-health format (such as a chat or a video consultation), as the development and testing of these e-health programs as a sole modality has been recommended [40].

It would also be important to include other dimensions of the TPB, namely, subjective norms and control beliefs, in the recommendation system before running the RCT. For instance, subjective norms may explain the parental underestimation of their child’s weight status and, eventually, the rejection of this reality after receiving this information [41]. Parental overweight status was also seen as a determinant to this underestimation [37], as parents may deny the idea of their children’s excessive weight to avoid having to take action on their own excessive weight [42]. It is also important to recognize the need to promote patient-centered communication rather than weight-focused communication in order to decrease the parent’s feelings of blame and to improve adherence and motivation while avoiding weight stigma [43]. This may be important to consider in the enrollment protocol for the RCT.

This study has some strengths and limitations that deserve attention. As strengths, this recommendation system was evidence-based, comprehensively promoted different lifestyle guidelines, and included an automated tailoring process. Furthermore, the recommendation system was tested in a pilot study in a real context, within a health care center, following the procedures of an RCT. This design and the detailed description and control of each procedure [29] will contribute to the external validity of the study in the future. The major limitation of this study is the small sample size. Nevertheless, the number of participants has been reported as enough to estimate average values and variability in pilot studies with continuous variables [25]. Additionally, our tailoring system was based on self-reported measures, which are susceptible to social desirability response bias [28]. Thus, in the RCT, it would be important to have more accurate measures to be used as the cutoff for the recommendation system—for instance, using devices with accelerometers (e.g., smartwatches or fitness bands) to evaluate PA. Additionally, to tailor recommendations, the system could use more than parents’ beliefs and children’s behaviors. Parents want these programs to take into account their concerns, so the system could also include their reported needs or preferences [44]. In the future, a qualitative study may help with understanding those preferences and concerns. Due to service and time constraints, it was not possible to run this qualitative study before the development of the recommendation system. In the RCT, it may also be important to involve more than one adult of reference (such as grandparents) or other environments frequented by children (such as kindergartens) in order to collect additional information and to raise awareness regarding the guidelines for children’s age.

## 5. Conclusions

An evidence-based, tailored recommendation system was developed in order to target parents of children aged 3–6 years identified in health care centers as having overweight/obesity. A pilot test in a health care center was designed to test the procedures of the RCT. This study aimed to describe the effect of its use on changing parents’ knowledge about the guidelines related to children’s lifestyle, as this can be seen as a short-term outcome that represents a potential mechanism of efficacy of the designed intervention. This study aimed to indicate potential adjustments before running the RCT.

Our study suggested that there is a need to work on parents’ motivation toward the desire to change the condition of their child’s weight status with additional strategies to those considered in the study. Providing knowledge to parents of preschool children about their child’s excessive weight or explaining the consequences of having too much weight at this age is not enough to motivate them to adhere to an e-health lifestyle intervention. Therefore, it would be important to promote patient-centered communication rather than weight-focused communication in order to decrease the parent’s feelings of blame and to improve adherence to the intervention. In the RCT, it would also be important to consider other strategies to change parents’ perception of their child’s excessive weight, for instance, by including one session of motivational interviewing. Additionally, a qualitative study may help with understanding parents’ preferences and concerns and give important feedback to improve recruitment.

This tailored recommendation system improved parent’s knowledge about the guideline for water intake, which was the least-known lifestyle guideline. However, a high percentage of parents that were already informed about the guidelines had failed to meet those guidelines. Thus, there is also a need to promote more than knowledge to help parents change their child’s lifestyle, as knowledge is not the only determinant of behavior. In the RCT, it will be possible to test the effect of recommendations related to food parenting practices on children’s eating behavior. Additionally, it may also be important to involve other caregivers of children or contexts to exert a higher influence on behavioral changes.

## Figures and Tables

**Table 1:** Aims for Each Lifestyle Component (Based on the Theory of Planned Behavior).

	Behavioral Beliefs^1^	Attitudes toward the Behavior	Perceived Behavioral Control
**Weight Status**	Parents are aware of their children’s excessive weight.	Parents are concerned about their children’s excessive weight.	Parents feel confident about improving their children’s excessive weight.
**Food and Beverage Intake**	Parents know that their children consume less fruit and vegetables, more energy-dense foods, more sugar-sweetened beverages, and less water than recommended.	Parents believe it is important that their children eat and drink as recommended.	Parents feel confident about improving their children’s intake.
**Physical Activity and Sleep**	Parents are aware that their children move less and sleep less than recommended.	Parents recognize that it is important that children move enough.	Parents feel confident about improving their children’s sleeping time.

^1^ The theory of planned behavior model also included normative beliefs and control beliefs, which were not included in this first version of the recommendation system.

**Table 2:** Questionnaire Developed to Tailor the Recommendations with Cutoffs to Receive a Recommendation Based on Different Dimensions of the Theory of Planned Behavior.

	Questions		Cutoff	Recommended Content TPB
**1. Weight Status**	(a) How do you classify the weight of your child for their age, sex, and height?^1^		≤5	Behavioral Beliefs
	(b) How concerned are you about your child’s weight status?^2^		≤5	Attitudes
**2. Food and Beverage Intake**	(a) What are the guidelines for your child’s age?^3^	Fruits (F) (portions per day)	F + V<5	Behavioral Beliefs
		Vegetables (V) (portions per day)		
		Energy-dense foods (portions per week)	>3	
		Water (glasses per day)	<8	
		Sugar-sweetened beverages (glasses per week)	>3	
	(b) What is the usual intake of your child?^3^	Fruits (F) (portions per day)	F + V<5	Attitudes and Perceived Behavioral Control
		Vegetables (V) (portions per day)		
		Energy-dense foods (portions per week)	>3	
		Water (glasses per day)	<8	
		Sugar-sweetened beverages (glasses per week)	>3	
**3. Physical Activity and Sleep**	(a) What are the guidelines for your child’s age?^4^	Moderate to intense PA (periods of 20 min of active play, speed walking, or any sport per day)	<3	Behavioral Beliefs
		Sleep (hours of nighttime sleep and napping per day)	<10	
	(a) How long does your child usually spend on each of these activities?^4^	Moderate to intense PA (periods of 20 min of active play, speed walking, or any sport per day)	<3	Attitudes and Perceived Behavioral Control
		Sleep (hours of nighttime sleep and napping per day)	<10	

^1^ Rating scale 0–10: 0—too slim; 10—too heavy; 5—healthy; ^2^ Rating scale 0–10: 0—not concerned at all; 10—extremely concerned; ^3^ Rating scale 0–10: in “portions” for fruits, vegetables, and energy-dense foods and “glasses” for water and sugar-sweetened beverages; ^4^ Rating scale 0–10: in “periods of 20 min” for moderate to intense PA and “hours” for sleep; TPB—theory of planned behavior; F—fruit; V—vegetables; PA—physical activity.

**Table 3:** Distribution of Videos by Lifestyle Component and Dimensions of the Theory of Planned Behavior.

	Theory of Planned Behavior			Food Parenting Practices Map
	Behavioral Beliefs^1^	Attitudes^2^	Perceived Behavioral Control^2^	
**Weight Status**	Video 1Healthy Development	Video 2Consequences of having overweight	Video 3How to help a child return to a healthy weight^3^	↓ Weight Talk↑ Guided Choices↑ Modeling
**Food and Beverage Intake**	Video 45 portions of fruit and vegetables daily^4^	Video 5Why 5 portions of fruit and vegetables a day?^3^	Video 6 Teach them to like, without pressure	↓ Pressure to Eat↑ Availability and Accessibility of Healthy Foods↑ Attractive Presentation of Foods↑ Nutrition Education↑ Modeling↑ Monitoring↑ Rules and Limits↓ Distractions↑ Availability and Accessibility
			Video 7Eating variety: Why does it matter?	
	Video 8 Addictive foods^4^	Video 9 How to recognize addictive foods^3^	Video 10 How to regulate the intake of addictive foods	
			Video 11 Distractions while eating	
	Video 12Hydration^4^		Video 13How to select a juice	
**Physical Activity and Sleep**	Video 14Move^4,5^			
	Video 15Sleep^4,6^			

^1^ Recommended if parents did not know or recognize their child’s excessive weight/the guidelines; ^2^ Recommended if children were not meeting the guidelines (with the exception of ^3^, which were “obligatory videos” recommended to all parents); ^4^ Priority videos; ^5^ Also focused on attitudes; ^6^ Also focused on perceived behavioral control.

**Table 4:** Characteristics of Parents/Caregivers and Children in Study 1 and Study 2 (Baseline).

		Study 1 (n = 35)	Study 2	
			Intervention Group (n = 11)	Control Group (n = 10)
**Parents/Caregivers**				
Sex	Female, n (%)	31 (88.6)	7 (63.6)	8 (80.0)
	Male, n (%)	4 (11.4)	4(36.4)	2 (20.0)
Age (years), mean (SD)		35.2 (4.6)	36.9 (3.1)	38.8 (3.7)
With university degree, n (%)		29 (82.8)	8 (72.7)	6 (60.0)
**Children**				
Sex	Female, n (%)	17 (48.6)	2 (18.2)	3 (30.0)
	Male, n (%)	18 (51.4)	9 (81.8)	7 (70.0)
Age (years), mean (SD)		4.4 (1.1)	4.9 (1.1)	5.2 (1.2)
Weight status, n (%)	Underweight	0 (0.0)	n.a.	n.a.
	Normal weight	25 (71.4)	n.a.	n.a.
	Overweight	7 (20.0)	6 (54.5)	4 (40.0)
	Obesity	3 (8.6)	5 (45.5)	6 (60.0)

n.a. = not applicable.

**Table 5:** Parents’ Eligibility to Receive Recommendations, According to the Answers to the Questionnaire and Considering the Cut-Off Selected to Receive a Recommendation (n = 35).

	Questions		Cutoff	Parents Eligible to Receive Recommendationsn (%)
**1. Weight Status^1^**	(a) How do you classify the weight of your child for their age, sex, and height?^2^		≤5	7 (70.0)
	(b) How concerned are you about your child’s weight status?^3^		≤5	6 (60.0)
**2. Food and Beverage Intake**	(a) What are the guidelines for your child’s age?^4^	Fruits (F) (portions per day)	F + V<5	13 (37.1)
		Vegetables (V) (portions per day)		
		Energy-dense foods (portions per week)	>3	1 (2.9)
		Water (glasses per day)	<8	30 (85.7)
		Sugar-sweetened beverages (glasses per week)	>3	0 (0.0)
	(b) What is the usual intake of your child?^4^	Fruits (F) (portions per day)	F + V<5	20 (57.1)
		Vegetables (V) (portions per day)		
		Energy-dense foods (portions per week)	>3	20 (57.1)
		Water (glasses per day)	<8	32 (91.4)
		Sugar-sweetened beverages (glasses per week)	>3	9 (25.7)
**3. Physical Activity and Sleep**	(a) What are the guidelines for your child’s age?^5^	Moderate to intense PA (periods of 20 min of active play, speed walking, or any sport per day)	<3	5 (14.3)
		Sleep (hours of nighttime sleep and napping per day)	<10	14 (40.0)
	(a) How long does your child usually spend on each of these activities?^5^	Moderate to intense PA (periods of 20 min of active play, speed walking, or any sport per day)	<3	9 (25.7)
		Sleep (hours of nighttime sleep and napping per day)	<10	16 (45.7)

F—fruit; V—vegetables; PA—physical activity;^1^ For the 10 children with overweight or obesity; ^2^ Rating scale of 0–10: 0—too slim; 10—too heavy; 5—healthy; ^3^ Rating scale of 0–10: 0—not concerned at all; 10—extremely concerned; ^4^ Rating scale of 0–10: in “portions” for fruits, vegetables, and energy-dense foods and “glasses” for water and sugar-sweetened beverages; ^5^ Rating scale of 0–10: in “periods of 20 min” for moderate to intense PA and “hours” for sleep.

**Table 6:** Change in Parents’ Perceptions about Weight and Knowledge about the Guidelines, Represented by Post-Test Scores and Differences Compared to the Baseline Test, for the Control and Intervention Groups (n = 15).

	Cutoff	Control (n = 8) Median (IQR)		Intervention (n = 7) Median (IQR)		Differences between Groups^1^		
		Post-Test	Dif.	Post-Test	Dif.	U	*p*-Value	*r^2^* (effect size)
Perceptions about the child’s excessive weight^2^	≤5	5.5 (1.8)	0.0 (2.0)	6.0 (1.0)	0.0 (1.0)	28.0	0.976	0.00
Fruits and vegetables (portions/day)^3^	<5	5.0 (1.75)	0.5 (3.0)	5.0 (3.0)	2.0 (3.0)	19.0	0.336	0.08
Water (glasses/day)^3^	<8	5.0 (3.0)	0.0 (2.0)	8.0 (1.0)	3.0 (2.0)	0.0	<0.001***	0.78

^1^ To compare the change between the control and intervention groups, the Mann–Whitney U test was used; ^2^ How do you classify the weight of your child for their age, sex, and height? (Responses on a 0–10 rating scale: 0—too slim; 10—too heavy; 5—healthy); ^3^ What are the guidelines for your child’s age? (Responses on a 0–10 rating scale, in “portions” for fruits and vegetables and “glasses” for water); IQR—interquartile range; Dif.—difference compared to baseline test; *r*^2^—coefficient of determination ***p < 0.001.
